# Supplementary material for: Antibacterial Effect of 16 Essential Oils and Modulation of mex Efflux Pumps Gene Expression on Multidrug-Resistant Pseudomonas aeruginosa Clinical Isolates: Is Cinnamon a Good Fighter?
Source: Antibiotics (Basel). 2023 Jan 12;12(1):163. doi: 10.3390/antibiotics12010163 (PMC9854426; doi:10.3390/antibiotics12010163)
Supplement: Supplementary file 1 [file antibiotics-12-00163-s001.zip › antibiotics-2151200-supplementary.pdf]

Table S1. Details on MIC and MBC values.

	Cinnamon			Thyme			Turmeric			Peppermint			Basil			Clove			Lavender		
**Sample number**	KB Ø	MIC %	MBC %	KB Ø	MIC %	MBC %	KB Ø	MIC %	MBC %	KB Ø	MIC %	MBC %	KB Ø	MIC %	MBC %	KB Ø	MIC %	MBC %	KB Ø	MIC %	MBC %
**1**	26	<0.0125	<0.0125	6	-	-	6	-	-	6	-	-	6	-	-	10	>25	>25	6	-	-
**2**	21	<0.0125	<0.0125	6	-	-	6	-	-	6	-	-	6	-	-	6	-	-	6	-	-
**3**	19	<0.0125	<0.0125	20	0.39	1.56	6	-	-	6	-	-	11	0.2	0.78	6	-	-	6	-	-
**6**	23	0.05	0.05	14	12.5	12.5	6	-	-	6	-	-	6	-	-	9	-	-	6	-	-
**8**	16	<0.0125	<0.0125	11	>25	>25	6	-	-	6	-	-	6	-	-	6	-	-	6	-	-
**9**	19	<0.0125	<0.0125	9	-	-	6	-	-	6	-	-	6	-	-	6	-	-	6	-	-
**12**	25	<0.0125	<0.0125	13	>25	>25	6	-	-	6	-	-	6	-	-	6	-	-	17	1.56	6.25
**13**	26	<0.0125	<0.0125	17	>25	>25	6	-	-	6	-	-	6	-	-	6	-	-	6	-	-
**14**	17	0.025	0.025	15	>25	>25	6	-	-	6	-	-	6	-	-	6	-	-	6	-	-
**15**	25	0.025	0.025	6	-	-	6	-	-	6	-	-	6	-	-	6	-	-	6	-	-
**16**	18	0.05	0.05	6	-	-	6	-	-	6	-	-	6	-	-	6	-	-	6	-	-
**18**	30	0.05	0.05	13	>25	>25	6	-	-	6	-	-	6	-	-	6	-	-	6	-	-
**20**	22	<0.0125	<0.0125	15	>25	>25	6	-	-	6	-	-	20	>25	>25	6			6	-	-
**21**	22	0.025	0.025	6	-	-	6	-	-	6	-	-	6	-	-	6	-	-	6	-	-
**22**	25	<0.0125	<0.0125	5	-	-	6	-	-	6	-	-	6	-	-	6	-	-	6	-	-
**23**	26	0.05	0.05	15	-	-	6	-	-	6	-	-	6	-	-	6	-	-	6	-	-
**24**	30	<0.0125	<0.0125	20	>25	>25	6	-	-	6	-	-	6	-	-	6	-	-	6	-	-
**26**	50	<0.0125	<0.0125	6	-	-	6	-	-	6	-	-	6	-	-	6	-	-	6	-	-
**27**	26	0.05	0.05	42	>25	>25	6	-	-	17	>25	>25	11	0.39	1.56	12	>25	>25	6	-	-
**28**	25	0.05	0.05	17	>25	>25	6	-	-	6	-	-	6	-	-	6	-	-	6	-	-
**29**	23	0.05	0.05	19	>25	>25	6	-	-	6	-	-	6	-	-	6	-	-	6	-	-
**30**	24	0.05	0.05	6	-	-	6	-	-	6	-	-	6	-	-	11	>25	>25	6	-	-
**31**	28	0.05	0.05	6	-	-	6	-	-	6	-	-	6	-	-	6	-	-	6	-	-

**32**	32	0.05	0.05	9	-	-	6	-	-	6	-	-	6	-	-	6	-	-	6	-	-
**33**	19	0.025	0.025	6	-	-	6	-	-	6	-	-	6	-	-	6	-	-	6	-	-
**34**	22	<0.0125	<0.0125	6	-	-	6	-	-	6	-	-	6	-	-	6	-	-	6	-	-
**36**	30	0.025	0.025	6	-	-	6	-	-	6	-	-	6	-	-	6	-	-	6	-	-
**38**	21	<0.0125	<0.0125	6	-	-	6	-	-	6	-	-	6	-	-	6	-	-	6	-	-
**39**	19	<0.0125	<0.0125	6	-	-	6	-	-	6	-	-	6	-	-	6	-	-	6	-	-
**41**	18	<0.0125	<0.0125	6	-	-	6	-	-	6	-	-	6	-	-	6	-	-	6	-	-
**44**	28	<0.0125	<0.0125	6	-	-	6	-	-	6	-	-	6	-	-	6	-	-	6	-	-
**47**	21	0.025	0.025	6	-	-	6	-	-	6	-	-	6	-	-	11	>25	>25	6	-	-
**48**	28	<0.0125	0.05	6	-	-	6	-	-	6	-	-	6	-	-	6	-	-	6	-	-
**49**	24	<0.0125	0.2	6	-	-	6	-	-	6	-	-	6	-	-	6	-	-	6	-	-
**50**	25	<0.0125	<0.0125	6	-	-	6	-	-	6	-	-	6	-	-	6	-	-	6	-	-
**51**	25	<0.0125	<0.0125	6	-	-	6	-	-	6	-	-	6	-	-	6	-	-	6	-	-
**52**	27	0.025	0.025	6	-	-	6	-	-	6	-	-	6	-	-	10	>25	>25	6	-	-
**53**	26	0.025	0.025	26	>25	>25	6	-	-	6	-	-	6	-	-	10	>25	>25	6	-	-
**56**	24	0.05	0.05	6	-	-	6	-	-	6	-	-	6	-	-	6	-	-	6	-	-
**58**	21	0.05	0.025	14	>25	>25	6	-	-	6	-	-	6	-	-	6	-	-	6	-	-
**59**	22	0.05	<0.025	6	-	-	6	-	-	6	-	-	6	-	-	6	-	-	6	-	-
**60**	22	0.05	0.05	6	-	-	6	-	-	6	-	-	6	-	-	6	-	-	6	-	-
**61**	24	0.025	0.025	6	-	-	6	-	-	6	-	-	6	-	-	6	-	-	6	-	-
**62**	17	0.05	0.025	6	-	-	6	-	-	6	-	-	6	-	-	6	-	-	6	-	-
**64**	26	0.05	0.05	6	-	-	6	-	-	6	-	-	6	-	-	6	-	-	6	-	-
**67**	24	<0.0125	<0.0125	22	1.56	1.56	6	-	-	6	-	-	6	-	-	6	-	-	6	-	-
**68**	21	<0.0125	0.025	16	>25	>25	6	-	-	6	-	-	6	-	-	6	-	-	6	-	-
**71**	14	0.025	0.025	6	-	-	6	-	-	6	-	-	6	-	-	6	-	-	6	-	-
**72**	30	<0.0125	0.05	6	-	-	6	-	-	6	-	-	6	-	-	6	-	-	6	-	-
**73**	18	<0.0125	<0.0125	6	-	-	6	-	-	6	-	-	14	0.2	0.78	6	-	-	6	-	-

**74**	16	<0.0125	<0.0125	6	-	-	6	-	-	6	-	-	6	-	-	6	-	-	6	-	-
**75**	30	<0.0125	<0.0125	17	>25	>25	6	-	-	6	-	-	6	-	-	6	-	-	6	-	-
**76**	26	0.05	0.05	24	>25	>25	6	-	-	6	-	-	6	-	-	6	-	-	12	>25	>25
**77**	28	0.05	0.05	20	>25	>25	6	-	-	12	>25	>25	6	-	-	6	-	-	6	>25	>25
**78**	24	0.025	<0.0125	6	-	-	6	-	-	6	-	-	6	-	-	6	-	-	8	-	-
**80**	32	<0.0125	<0.0125	8	-	-	6	-	-	6	-	-	6	-	-	6	-	-	6	-	-
**82**	26	<0.0125	0.05	6	-	-	6	-	-	6	-	-	6	-	-	6	-	-	6	-	-
**83**	18	<0.0125	<0.0125	6	-	-	6	-	-	6	-	-	6	-	-	6	-	-	6	-	-
**84**	48	<0.0125	<0.0125	50	>25	>25	11	12.5	12.5	23	0.2	0.2	11	0.39	0.39	6	-	-	11	0.39	0.39
**88**	26	<0.0125	<0.0125	17	>25	>25	6	-	-	6	-	-	6	-	-	6	-	-	6	-	-
**90**	24	0.025	0.025	6	-	-	6	-	-	6	-	-	6	-	-	6	-	-	6	-	-
**102**	24	<0.0125	<0.0125	6	-	-	6	-	-	6	-	-	6	-	-	6	-	-	12	>25	>25
**128**	25	0.025	0.025	30	>25	>25	6	-	-	6	-	-	6	-	-	6	-	-	6	-	-
**141**	30	0.05	0.05	6	-	-	6	-	-	6	-	-	6	-	-	6	-	-	14	>25	>25
**142**	19	0.025	0.025	6	-	-	6	-	-	6	-	-	6	-	-	6	-	-	6	-	-
**145**	24	0.05	0.05	20	>25	>25	6	-	-	6	-	-	6	-	-	6	-	-	6	-	-
**150**	44	<0.0125	<0.0125	38	>25	>25	6	-	-	6	-	-	13	0.2	0.2	6	-	-	13	0.05	0.05
**151**	26	0.025	0.025	27	>25	>25	6	-	-	6	-	-	6	-	-	6	-	-	6	-	-
**152**	25	<0.0125	<0.0125	6	-	-	6	-	-	6	-	-	6	-	-	6	-	-	6	-	-
**153**	21	0.05	0.05	20	>25	>25	6	-	-	12	>25	>25	6	-	-	6	-	-	11	>25	>25
**154**	20	0.05	0.05	26	>25	>25	6	-	-	6	-	-	6	-	-	6	-	-	12	6.25	6.25
**160**	30	0.025	0.025	6	-	-	6	-	-	6	-	-	6	-	-	6	-	-	6	-	-

KB Ø- Kirby Bauer method diameter (millimeters); MIC- minimal inhibitory concentration; MBC-minimum bactericidal concentration.
